# Etiology of Urethral Inflammation

**Published:** 1883-07

**Authors:** Henry J. Reynolds

**Affiliations:** Orion, Mich.


### ARTICLE VI.

ETIOLOGY OF URETHRAL INFLAMMATION. By HENRY J. REYNOLDS, M. D., Orion, Mich. (Read before the Michigan State Medical Society, at Kalamazoo, in May, 1883.)

The value of a careful investigation and correct knowledge of the etiology of individual cases of urethral inflammation cannot be overestimated. It is not only a very important matter with a view to the intelligent treatment of each case, but also in a moral or medico-legal sense, with a view to justice to the individual so affected; to justice between the persons so affected and the person concerned in the supposed source of contagion; and still further, we can imagine a case where it would involve the question of justice between man and wife.

Though, in a great majority of cases, urethral inflammation may be ascribed to one principal cause, viz: that of infection from the virus of the specific form of disease known as gonorrhœa, yet there are numerous other causes giving rise to a form of disease not clinically differing from true gonorrhœa, and to all appearance almost identical with it; both being inflammations of the urethra, associated with the discharge of pus or mucopus, as the case may be, and having no diagnostic features by which the one may be differentiated with certainty from the other. No intelligent physician will doubt for a moment the possibility, at least, of having a discharge from the urethra, with all the accompanying symptoms of true gonorrhœa, when the disease, nevertheless, may have arisen from some cause other than that of contact with the specific virus of gonorrhœa, and still, I think it is a too common practice with physicians in general, where the cause is obscure, to ascribe the disease to contact, during the sexual act, with a diseased person of the opposite sex. There is no doubt that many an innocent person has been wrongfully accused, and thereby seriously injured in this respect, and as Van Buren has said, "it is better that a hundred of the guilty should escape, than that one innocent person should be accused."

The various forms of urethral inflammation may be summed up under two principal heads, viz:—simple urethritis, and specific or virulent urethritis.

By the term simple urethritis, I would embrace all those forms of urethral inflammation not caused or produced by contact with what is known as gonorrhœal virus; these various forms differing only in their etiology and extent, and not in the quality of the inflammatory action.

This inflammation, under favorable circumstances, usually runs a short, mild course, and may be produced by almost any kind of irritation, such as, for instance, a foul, acrid or decomposing menstrual or leucorrhœal discharge; strongly acid or concentrated urine; injury by the rough use of urethral instruments; passage of stone fragments or quantities of large uric acid crystals; irritation to the perineum, as in horseback exercise, riding on a wagon without springs, etc. Any of the above causes are capable of producing a simple urethritis, no doubt, but are more particularly so when combined with pre-existing stricture, idiosyncrasy, uncleanliness, impaired urethral tone, as from sexual excess, etc. Inflammation produced by any of these causes, though tending to run a mild course and get well rapidly, will, under unfavorable circumstances, frequently remain very stubborn and unyielding to treatment.

A simple urethritis from any cause, once fully established, can almost invariably be aggravated by the use of too strong injections, together with acrid or irritating urine, perineal irritation and general dissipation. In such a case, it may assume fully as virulent a form as true gonorrhœa from contagion, leaving all the evil consequences, as gleet, stricture, etc., and may even, in my opinion, contain the necessary contagious elements to impart disease readily to others by contact with the same.

The mildest form of a simple mucous catarrh from the meatus may be ultimately aggravated into a most virulent urethritis by the indiscreet use of a too strong injection in an unusually sensitive urethra. I have seen the inflammation greatly increased by the injection of a cheap insoluble form of sulphate of zinc, some of the crystals remaining undissolved, and being carried into the urethra in that state. Other cases I have seen greatly aggravated by simply riding on the cars; the warmth from the cushioned seat and the constant jarring motion of course being the cause.

Hence, a man may acquire, without sexual, or even "water-closet" exposure, a disease almost, if not entirely identical with true gonorrhœa from contagion; the only difference, if any, between which and true gonorrhœa being that it is *possibly* devoid of the elements of contagion.

All physicians are no doubt familiar with the contagious character of purulent conjunctivitis; even a catarrhal form of conjunctivitis may be transmitted from one to another by contagion. Both of these diseases, it is generally, if not universally conceded, may arise spontaneously; that is, entirely independently of contagious influences. Here, then, is an inflammation of a mucous membrane developing spontaneously, as it were, into a violent, contagious disease. Now, it seems highly probable that the same thing may and does take place in the mucous membrane of the urethra, and hence may be developed, without contagion, a disease possessing all the distinguishing characteristics of true gonorrhœa. The disease is now no longer simple urethritis, but has become developed, either in the same individual or through a series of inoculations, into a specific, virulent, or contagious urethritis.

The following cases, the veracity of the statements and history in each case being, to my mind, beyond a question, furnish illustrations of simple urethritis, or urethritis without contagion.

Case I. F. C., commercial traveler, æt. thirty, with good constitution and somewhat dissipated habits, came under my care for gleet in 1879. Examined and found stricture, for which patient declined submitting to any operative treatment. The discharge continued more or less until the summer of 1880, when it ceased altogether. In the fall of 1881, or more than a year after the discharge had disappeared, I was again consulted, patient stating that the old disease had come back on him. Stated also that he had positively not had any exposure to new disease, which was in all probability true, as he was engaged to be married soon. In this case the disease was evidently not due to gonorrhœal infection, but probably chiefly to the stricture.

Case II. R. J., æt. twenty-eight, driver of fast horses; good habits and good constitution; wears suspensory bandage, however, for bad varicocele, contracted in fall of 1880, though without having had any suspicious intercourse; a disease, to all appearances, true gonorrhœa.

The discharge continued in abundance for five weeks, and was followed by gleet, which lasted four weeks, and a little increased frequency of urination, which continued for a time after the gleet stopped. No examination made, but quite probably a small amount of stricture existed. In one year after the first attack, patient had another attack, which however, was more mild; not lasting over three or four weeks, and not followed by any gleet or other bad symptoms. In the following spring, a third attack set in. This attack was mild at first, but owing to riding on a sulkey without springs (which every time seemed to increase the trouble), violent exercise, etc., the disease lingered and grew gradually worse, and finally, after having run a very severe course, was followed by gleet which has lasted ever since. On September 14, 1882, six months from date of last attack, I examined and found stricture at two and one-half ($2\frac{1}{2}$) inches and at six and one-half ($6\frac{1}{2}$) inches from the meatus. It might be well to mention that there had been no suspicious intercourse previous to any of these attacks, and that the last intercourse of any kind prior to the last attack was at least twelve days before the appearance of the discharge. It is extremely probable, therefore, that the last two attacks, at least, in this case, were not the result of gonorrhœal contagion, but were produced by the following causes: stricture, resulting from first attack; the perineal irritation from riding the sulkey; the irritating pressure of the band of the suspensory bandage behind the scrotum upon the urethra; strongly acid urine, etc. The irritation from riding the sulkey and the pressure of the suspensory bandage both, however, the patient stated, increased the intensity of the disease when present.

Numerous other cases similar to the above might be quoted, would time and space permit, but these, which no doubt accord with the experience of many practitioners, may help to illustrate the point I wish to establish, viz., that we are, much more frequently than we readily concede, called upon to treat cases of urethral inflammation which we diagnosticate as true gonorrhœa, where the real disease is only a simple urethritis, being produced by some of the numerous causes outside of gonorrhœal contagion.

With regard to the cause of what I should designate the specific or virulent form of the disease, I will say but little in this paper. It is, of course, always said to be the result of contact with the propagating element of the discharge of the so-called true gonorrhœa. It seems probable in my mind, however, that a contagious, virulent, or specific form of disease may be developed from contact with the discharge from a violent form of simple urethritis, especially where uncleanliness and other circumstances favor the development of the same exist.

In conclusion I will add, that while, as a general thing, it makes little or no difference to the physical well-being of those affected whether all cases of simple urethritis be called by the name of, or even treated as gonorrhœa, I can imagine a case where such a course might be productive of great evil and damage, both to the patient and physician, and which would be in reality little short of, and possibly as actionable for damage as, real malpractice.

